# Characterization of Initial Parameter Information for Lifetime Prediction of Electronic Devices

**DOI:** 10.1371/journal.pone.0167429

**Published:** 2016-12-01

**Authors:** Zhigang Li, Boying Liu, Mengxiong Yuan, Feifei Zhang, Jiaqiang Guo

**Affiliations:** Department of Electrical Engineering, Hebei University of Technology, Tianjin, China; Beijing University of Technology, CHINA

## Abstract

Newly manufactured electronic devices are subject to different levels of potential defects existing among the initial parameter information of the devices. In this study, a characterization of electromagnetic relays that were operated at their optimal performance with appropriate and steady parameter values was performed to estimate the levels of their potential defects and to develop a lifetime prediction model. First, the initial parameter information value and stability were quantified to measure the performance of the electronics. In particular, the values of the initial parameter information were estimated using the probability-weighted average method, whereas the stability of the parameter information was determined by using the difference between the extrema and end points of the fitting curves for the initial parameter information. Second, a lifetime prediction model for small-sized samples was proposed on the basis of both measures. Finally, a model for the relationship of the initial contact resistance and stability over the lifetime of the sampled electromagnetic relays was proposed and verified. A comparison of the actual and predicted lifetimes of the relays revealed a 15.4% relative error, indicating that the lifetime of electronic devices can be predicted based on their initial parameter information.

## 1. Introduction

The lifetime of an electronic device is generally estimated by conducting a whole lifetime test on a batch of device samples to calculate the statistical reliability of these samples. However, the service life of the device cannot be estimated with this method. Lifetime prediction can contribute toward improving the operational reliability and system reliability of electronics. Several studies have been conducted to investigate two forms of product lifetime prediction [1–4]: model-based prediction and data-based prediction.

Lifetime prediction models can be divided into classical and online prediction models. A classical prediction model is an offline prediction model based on the generalization of the results of multiple tests [5–7]. For example, Fontana established a mathematical model to determine the relationship between the lifetime and operating parameters (load current, ambient temperature and operating frequency) of a relay by using these parameters as predictor variables and assuming the lifetime of the relay to follow the Weibull Distribution [8]. The Center for Advanced Life Cycle Engineering at the University of Maryland proposed life consumption monitoring (LCM) and, based on its analyses of the failure mechanisms [9] and modes [10] of electronics, established a model to analyze the fretting wear of the devices under various stress conditions of temperature, humidity, vibration, voltage, and current [11], and integrated data obtained from these different stress conditions with a model to identify the health states of the devices that predicted their residual lifetime [12, 13]. Online prediction models use mathematical theories to monitor the degradation of predictor variables in real-time and perform modeling [14–16]. Lu et al. applied LCM to measure the damage to electronics operating under various stress conditions and propose an optimized autoregressive model for lifetime prediction that accounted for the degradation of the devices and the effects of abrupt stress changes on prediction; however, the authors yielded inaccurate results at the early stage of prediction [17]. Based on the measurability of the super-path time and pick-up time of relays, Zhai et al. developed a time-series mathematical model that used both variables to predict the lifetime of electronics [18]. This model-based prediction method examines the physical characteristics of electrical systems to illustrate the nature of the systems and enable the real-time prediction of their lifetime. However, it fails to establish accurate mathematical models for complex dynamical systems and, when applied for engineering purposes, can only handle systems with accurate mathematical models.

Contrary to the aforementioned, data-based prediction methods have higher adaptability and operability and are extensively applied in studies on product lifetime prediction across the world [19–21]. However, because of their limited capability, existing data-based prediction methods predict electronics lifetimes largely based on the static contact resistance [22, 23]. For example, Yao et al. examined the time-varying pattern of contact resistance to classify the closing of contact points into steady, erratic, and upward states and determine the stability of these points [24]. The authors used contact resistance as a predictive parameter to develop an integrated prediction model for these different closed states of contact points, which successfully predicted the steady and upward changes in contact resistance on a short-term basis. Caesarendra et al. collected the real-trending data of low-methane compressors and used a state–space model and particle filtering to predict the operational degradation of the compressors, thereby validating a prognosis algorithm of particle filtering that they proposed for application in real dynamic systems [25]. Jin et al. utilized historical degradation data to perform degradation modeling [26]. They applied a particle filter-based state and static parameter joint estimation method to obtain an iteratively updating posterior degradation model [27] and predict the degradation state of individual batteries [28] in spacecrafts. Lin and Zhang established the relationships of furfural concentration and carbon and carbon dioxide volumes in an oil-immersed power transformer with the reliability, aging degree, and remaining lifetime range of its solid insulating materials to develop a back-propagation (BP) neural network that predicted the residual lifetime of the device [29]. The aforementioned studies, which examined the performance parameters of electronics over their lifetimes, used estimation methods to establish models explaining the relationship between the lifetime and the degradation of the parameters [30–32]. However, some of these parameters, when in application, may yield highly uncertain and incomplete data, which can add considerable difficulty to lifetime prediction.

The service life of electronic devices that are difficult to monitor constantly in real-time can be estimated only on the basis of their early performance parameter values, rather than data on their lifetime or performance degradation. These parameter values are referred to as initial parameter information, which is obtained before an electronic device is used or after it has begun its first-time operation for a set time. However, the potential defects of the device may exist in the initial parameter information and affect its lifetime to some extent. Initial parameter information that contains such defects can be identified and the values and stability of the parameters be quantitatively analyzed to model the relationship of parameter value and stability with lifetime, thereby providing a new approach to predict the lifetime of the device.

## 2. Characterization of initial parameter information

The operation of an electronic device is affected by multiple factors. Its parameters therefore exhibit some degree of uncertainty and dispersion and cannot accurately indicate its performance. Thus, this study defined the performance parameters of an electronic device that were obtained in its first-time operation for a set time as the initial parameter information of its performance.

**Fig 1:**
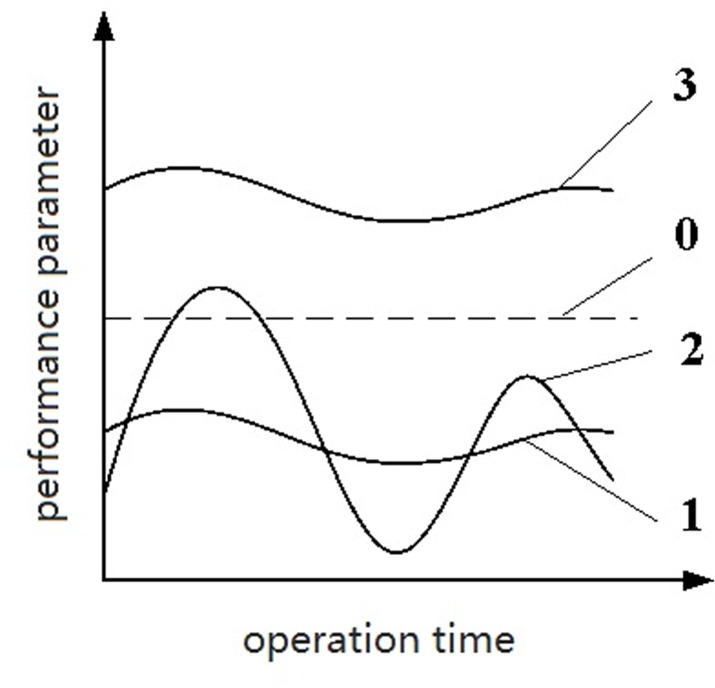
Diagram of the values of performance parameter and their stability.

Some electronics perform at their best with appropriate parameter values and low parameter stability. Thus, the performance of an electronic device, which reflects the levels of its potential defects, can be determined by the initial parameter information values and stability of its samples. An electronic device with samples for which initial parameter information values are closer to their optimal levels and have lower stability has lower levels of potential defects and a longer lifetime. Fig 1 depicts the relationship between initial parameter information value and stability.

In Fig 1, the dotted line 0 is the optimal level of a performance parameter; the solid lines 1 and 2 indicate almost the same parameter values but different levels of parameter stability, and line 1 is more stable than line 2. The solid lines 1 and 3 indicate almost the same levels of parameter stability but different parameter values, line 3 indicates exceeding the optimal level of a performance parameter, while the line 1 indicates not reach the optimal level of a performance parameter. Although they all deviate from the best running state, but the impact of actual operation are not same. The solid lines 2 and 3 indicate different parameter values and different levels of parameter stability. The performance of an electronic device depends on the distances of its performance parameters from their optimal levels and on the stability of these parameters.

### 2.1 Values of the initial parameter information

The initial parameter information constitutes a time series of performance parameters obtained from a newly manufactured electronic device that was operated for a set time. The performance parameters of the device were characterized by set degrees of randomness and uncertainty because of multiple factors affecting its operation.

To reduce the effects of randomness and uncertainty on parameter values, the weighted arithmetic mean was used to calculate the means of the initial parameter information of the samples, and the mean values were defined as the indicators of the parameters. The weight of each sample was defined by the relative probability density (PD) of its parameters, which was derived by estimating the probability density functions (PDFs) for these parameters. A high PD value indicates the high occurrence of an individual point and a high weight value.

#### 2.1.1 Estimation of probability density function

PD values derived using the rule-of-thumb estimation of density vary according to the partitioning of intervals by the method. Thus, kernel density estimation (KDE) was alternatively applied to estimate the PDFs for the initial parameter information [21].

That X = {x_1_,x_2_,⋯,x_n_} is the sample set, *n* is the number of samples, x_i_ is the *i*–th sample, x_i_ = {x_i1_,x_i2_,⋯,x_im_} is the set of the initial parameter information of the *i*–th sample, and *m* is the number of the initial parameter information of the *i*–th sample was supposed. Thus, the PDF for the initial parameter information of the *i*–th sample was defined as *f*_*i*_(*x*), and the KDE value ${\hat{f}}_{i}(x)$ for *f*_*i*_(*x*) at the random point x as

$${\hat{f}}_{i}(x){=}_{\bar{mh}}\sum_{j=1}^{m}K(\frac{x-x_{ij}}{h})$$ (1)

Where *h* is the window width or bandwidth and *K* is a kernel function. And *K* was chosen as the Gaussian kernel function, and *s* was the sample standard deviation with the optimal window width of *h* = 1.06*sn*^−0.2^.

**Fig 2:**
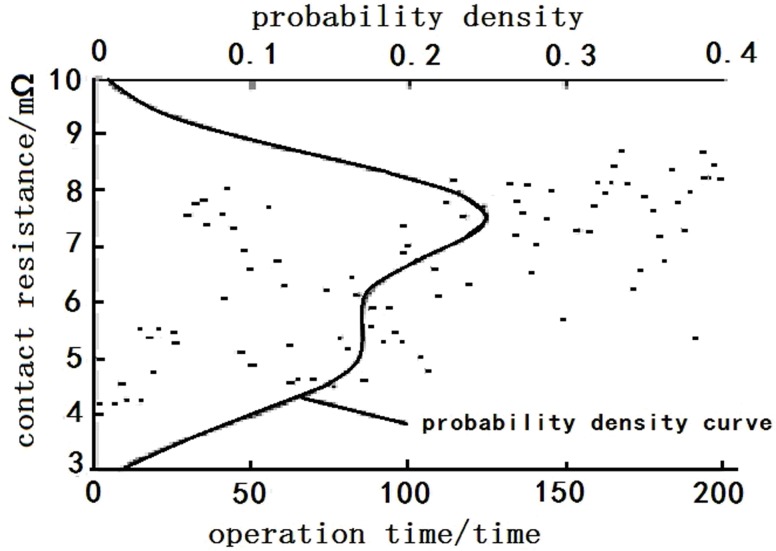
Diagram of initial parameter information’s probability density.

Fig 2 presents a PDF curve for the initial parameter information (contact resistance) of a sample relay, with the number of operations denoted by the x-axis and contact resistance by the y-axis. In this figure, “●” represents the level of contact resistance for the first 200 operations of the relay, and the PDF curve for the contact resistance was plotted through KDE.

#### 2.1.2 Weighted average probability of the initial parameter information

The weights and means of the initial parameter information for the *i*–th sample were derived using its PDF, as expressed by (2):

$$\left\{ \begin{array}{l} w(j)=f_{i}(x_{ij})/\sum_{j=1}^{m}f_{i}(x_{ij})\\ \bar{x}=\sum_{j=1}^{m}x_{ij}\times w_{i}(j) \end{array}\right.$$ (2)

Where *i* = 1,2,⋯,*n*, *w*_*i*_(*j*) is the weight of the *j*–th initial parameter information *x*_*ij*_ of the *i*–th sample, and ${\bar{x}}_{i}$ is the mean of the initial parameter information of the *i*–th sample.

### 2.2 Stability of the initial parameter information

To measure the stability of the initial parameter information, a least-squares polynomial fit [22] was performed with the raw data points of the parameters, and yielded a smooth fitting curve. This fitting curve, which showed the overall changes in the parameters, had multiple parameter values in the neighborhood of the extrema. Accordingly, the differences of the extrema and these adjacent parameters between them reflected the levels of the stability of the parameters.

#### 2.2.1 Deriving the extrema and end points

That *t*_*i*_ = {*t*_*i*1_,*t*_*i*2_,⋯,*t*_*im*_} was the set of measurement time periods for the set of the initial parameter information of the *i*–th sample x_i_ = {x_i1_,x_i2_,⋯,x_im_} was supposed. Thus, the set of discrete data points for these parameters was expressed by {(*t*_*ij*_,*x*_*ij*_), *j* = 1,2,⋯,*m*}.

When the sum of the square error between the polynomial function value $\hat{x}(t_{ij})$ at the point *t*_*ij*_(*j* = 1,2,⋯,*m*) and the original value *x*_*ij*_ was at its minimum, a k-polynomial function was used to fit the discrete data point set for the initial parameter information of the *i*–th sample and a k-polynomial function is derived, as expressed by (3):

$${\hat{x}}_{i}(t)=a_{i0}+a_{i1}t+a_{i2}t^{2}+\cdots +a_{ik}t^{k},(k<m)$$ (3)

Thus, the curve expressed by this polynomial function denoted the fitting results of the initial parameter information of the samples. Furthermore, the curve-fitting function was estimated to enable its derivative to satisfy (4):

$$d{\hat{x}}_{i}(t)/dt=0$$ (4)

The solutions of (4) were the x-axes of the extrema. The number of the solutions was (*k*−1). The solutions were arranged in value from the smallest to the largest: $\overset{*}{t_{i1}},\overset{*}{t_{i2}},\cdots ,{\overset{*}{t}}_{i(k-1)}$. Their corresponding extrema on the fitting curve were arranged as ${\hat{x}}_{i}({\overset{*}{t}}_{i1}),{\hat{x}}_{i}({\overset{*}{t}}_{i2}),\cdots ,{\hat{x}}_{i}({\overset{*}{t}}_{i(k-1)})$. The function values ${\hat{x}}_{i}(a)$ and ${\hat{x}}_{i}(b)$ of the fitting function ${\hat{x}}_{i}(t)$ respectively denotes the values of the interval endpoints *a* and *b* for the number of operations.

#### 2.2.2 Representation of parameter stability

That $y_{i}=\{{\hat{x}}_{i}(a),{\hat{x}}_{i}({\overset{*}{t}}_{i1}),{\hat{x}}_{i}({\overset{*}{t}}_{i2}),\cdots ,{\hat{x}}_{i}({\overset{*}{t}}_{i(k-1)}),{\hat{x}}_{i}(b)\}$ is the set of all extrema and end points for the initial parameter information of the fitting curve for the *i*–th sample, and the number of elements for the set *y*_*i*_ is k + 1. Thus, *y*_*i*_(*j*) was defined as the *j*–th element of the set *y*_*i*_. Hence, the difference between elements adjacent to the set *y*_*i*_ was estimated using (5):

$$\Delta y_{i}(j)=\left|y_{i}(j+1)-y_{i}(j)\right|(j=1,2,\cdots ,k)$$ (5)

Where max(*y*_*i*_) is the maximum of all the elements of the set *y*_*i*_ and min(*y*_*i*_) is the minimum of all the elements of the set *y*_*i*_. Hence, the maximum difference between all the extrema and end points on the y-axis of the fitting curve was estimated using (6):

$$\Delta y_{\max}=\max(y_{i})-\min(y_{i})$$ (6)

**Fig 3:**
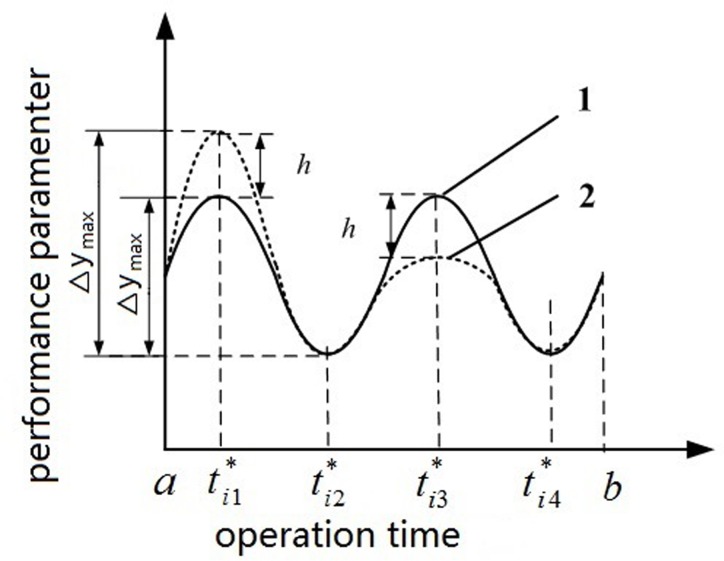
Diagram of performance parameter’s stability(a).

**Fig 4:**
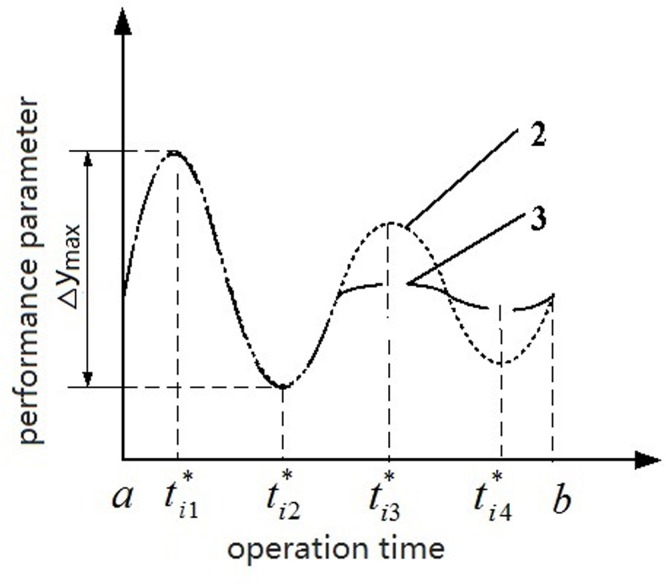
Diagram of performance parameter’s stability(b).

In Figs 3 and 4, curves (1), (2), and (3) present different levels of parameter stability. Notably, In Fig 3, curves (1) and (2) share almost the same total level of stability [namely, $\sum_{j=1}^{k}\Delta y_{1}(j)=\sum_{j=1}^{k}\Delta y_{2}(j)$], whereas the values for the maximum difference between the extrema are not identical (namely, Δ*y*_max_1_ ≠ Δ*y*_max_2_). In Fig 4, the values Δ*y*_max_ of curves (2) and (3) are almost identical, whereas the total levels of stability $\sum_{j=1}^{k}\Delta y_{i}(j)$ (i = 2, 3) in both curves are not same.

In sum, the stability of the initial parameter information was related to not only Δ*y*_*i*_(*j*), *j* = 1,2,⋯,*k* but to Δ*y*_max_. Thus, the weighted-average adjacent difference and maximum difference between end points and extrema were defined as the levels of parameter stability; larger differences were related to higher weight values of these differences. The weight *w*_*i*_(*j*) of the *j*–th difference for the *i*–th sample and the weight *w*_*i*_(k + 1) of Δ*y*_max_ were estimated using (7):

$$\left\{ \begin{array}{l} w_{i}(j)=\Delta y_{i}(j)/(\sum_{j=1}^{k}\Delta y_{i}(j)+\Delta y_{\max})\\ w_{i}(k+1)=\Delta y_{\max}/(\sum_{j=1}^{k}\Delta y_{i}(j)+\Delta y_{\max}) \end{array}\right.$$ (7)

The stability of the initial parameter information for the *i*–th sample was estimated using (8):

$${\tilde{y}}_{i}=\sum_{j=1}^{k}w_{i}(j)\times \Delta y_{i}(j)+(w_{i}(k+1)\times \Delta y_{\max})$$ (8)

## 3. Selection of the best wavelet packet basis

Based on their equations, the initial parameter information value was defined as the level of parameter value and the parameter stability was defined as the level of parameter variation. However, the orders of magnitude of parameter value and stability might differ from each other. To facilitate a comprehensive analysis of both indicators and neutralize the influence of the difference between their orders of magnitude, both indicators were normalized and their relationship with lifetime was subsequently modeled.

### 3.1 Normalization

When the performance of an electronic device is measured, parameters that perform better with higher values are defined as benefit parameters; parameters that perform better with lower values are defined as cost parameters; and parameters that perform well with moderate values are defined as moderate parameters. Respectively, I1, I2, and I3 denote the sets of benefit, cost, and moderate parameters.

The equation X = {*x*_1_,*x*_2_,⋯,*x*_*n*_} was defined as the sample set; *n* was defined as the number of samples; $\bar{X}=\{{\bar{x}}_{1},{\bar{x}}_{2},\cdots ,{\bar{x}}_{n}\}$ was defined as the set of parameter values in *n* samples; and $\tilde{y}=\{{\tilde{y}}_{1},{\tilde{y}}_{2},\cdots ,{\tilde{y}}_{n}\}$ was defined as the set of parameter stability levels in *n* samples. Hence, the normalized parameter value *x*_*g*_(*i*) of the *i*–th sample was estimated using (9):

$$x_{g}(i)=\left\{ \begin{array}{l} (x_{c1}-{\bar{x}}_{i})/(x_{c1}-{\bar{x}}_{\min})\;x_{i}\in I_{1}\\ (x_{i}-x_{c2})/({\bar{x}}_{\max}-x_{c2})\;x_{i}\in I_{2}\\ \left|{\bar{x}}_{i}-x_{c3}\right|/\max(\left|{\bar{x}}_{\max}-x_{c3}\right|,\left|{\bar{x}}_{\min}-x_{c3}\right|)\;x_{i}\in I_{3} \end{array}\right.$$ (9)

Where *i* = 1,2,⋯,*n* and *x*_*c*1_, *x*_*c*2_, *x*_*c*3_ represent the optimal values for *I*_1_, *I*_2_ and *I*_3_, respectively; and ${\bar{x}}_{\max}$ and ${\bar{x}}_{\min}$ are the maximum and minimum of the set $\bar{x}$

The normalized parameter values yg(*i*) of the *i*–th sample were estimated using (10):

$$y_{g}(i)=(y_{i}-y_{c})/({\tilde{y}}_{\max}-y_{c})$$ (10)

Where *i* = 1,2,⋯,*n*; ${\tilde{y}}_{\max}$ is the maximum of the set $\tilde{y}$; and *y*_*c*_ is the reference value of parameter stability when the device operated at its highest performance.

When an electronic device operates at its highest performance, the performance parameters *x*_*c*1_, *x*_*c*2_, and *x*_*c*3_ and the reference value *y*_*c*_ should be specified on the basis of its design parameters. Furthermore, after parameter value and stability were normalized using (9) and (10) respectively to [0, 1], the closer the values of both indicators were to 1, the more poorly the device performed, whereas the closer these values were to 0, the more efficiently the device performed.

### 3.2 Data modeling

The equation *x*_*g*_ = {*x*_*g*_(1),*x*_*g*_(2),⋯,*x*_*g*_(*n*)} was defined as the sample set, *x*_*g*_ = {*x*(1),*x*(2),⋯,*x*(*n*)} as the set of normalized parameter values in *n* samples, y_g_ = {*y*_*g*_(1),*y*_*g*_(2),⋯,*y*_*g*_(*n*)} as the set of normalized parameter stability levels in *n* samples, and *T*_*g*_ = {*T*_1_,*T*_2_,⋯,*T*_*n*_} as the set of the actual lifetime periods of *n* samples.

**Fig 5:**
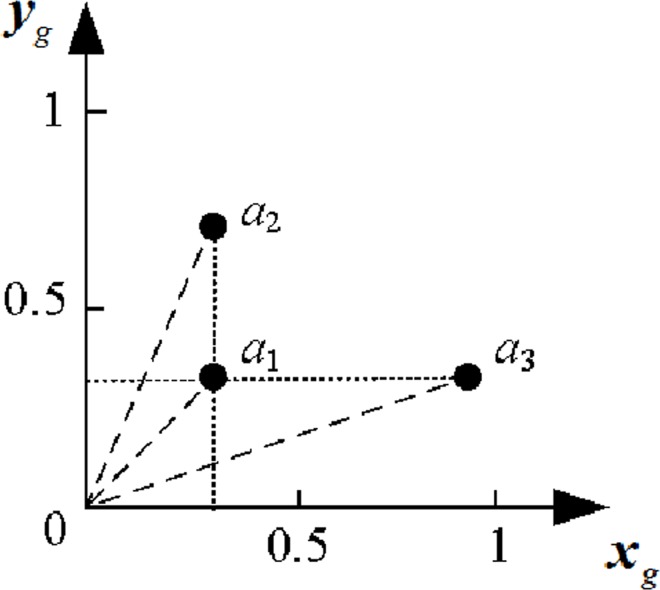
Diagram of the values of initial parameter information’s two indicators.

When *n* is large, artificial intelligence algorithms such as BP neural networks can be used to model the relationship of *x*_*g*_ and *y*_*g*_ with lifetime T. Details about modeling algorithms are referred to in [33]. When *n* is small, a criterion should be developed and the relationship between the criterion and lifetime should be established, instead of using artificial intelligence algorithms. And Fig 5 illustrates the possible distributions of two criteria for the initial parameter information, with *x*_*g*_ being the horizontal axis and *y*_*g*_ the vertical axis.

In Fig 5, *a*_1,_*a*_2_ and *a*_3_ denote the distributions of normalized parameter means and volatilities of three different samples. The distributions of *a*_1_ and *a*_2_ on the x-axis are identical, as well as those of *a*_1_ and *a*_3_ on the y-axis. This indicates that *a*_1_ performed more efficiently than *a*_2_ and *a*_3_ did but that the performance of *a*_2_ and *a*_3_ could not be determined. Thus, the criteria of samples were obtained by estimating the weighted distance between the data points and origin in the samples, as expressed by (11):

$$d_{i}=\sqrt{\alpha \cdot x_{b}^{2}(i)+\beta \cdot y_{b}^{2}(i)}$$ (11)

Where *α* and *β* are weighting factors, and *α* + *β* = 1. The values of *α* and *β* depended on the ration of *x*_*b*_(*i*)/*y*_*b*_(*i*), which was determined by the relative contribution of initial parameter value and stability to the performance of electronic devices. If the initial parameter information value contributed more than initial parameter information stability did to device performance, then *α* > *β*. If initial parameter information stability contributed more than initial parameter information value did to device performance, then *α* < *β*. If both equally contributed to device performance, then *α* = *β* = 0.5.

Based on the definition of criterion, the function model of criterion and device lifetime is established, as expressed by (12):

T=f(d) (12)

Where T is device lifetime and *d* is the criterion of a device.

## 4. Case analysis

Contact resistance is one of the main performance indicators for electromagnetic relays, and when it is low and stable, the electronic devices perform optimally. Accordingly, this study defined the contact resistance for the first 1000 operations of electromagnetic relays as the initial contact resistance of the relays.

A whole lifetime test was performed on eight samples relays to obtain their individual lifetimes. The contact resistance after each closing of the contact point was measured.

The initial contact resistances and parameter stability of the samples were quantified to model the relationship between these values and the lifetime of the samples:

1. The PDFs for the initial contact resistance of all the samples were derived using KDE, and (2) was applied to derive the means of the initial contact resistance of the samples.
2. Curve fitting was performed on the initial contact resistance of the samples to derive the extrema and end points, and (5), (6), and (7) were used to derive parameter stability.
3. The contact resistance of the samples was a cost parameter; thus, *x*_*c*2_ = 0 and *y*_*c*_ = 0, and (9) and (10) were used to derive the normalized parameter values and stability.
4. Because of the limited sample size, a modeling algorithm was employed to establish lifetime prediction models for the samples. Furthermore, with *α* = 0.7 and *β* = 0.3, criteria for the samples were obtained using (11). Table 1 tabulates the actual lifetime and initial contact resistance of each sample.
5. The relational function for the criterion d and lifetime T (unit: 10,000 times) for each relay that were obtained using polynomial fitting was expressed by (13):

$$T=-609.4d^{3}+1242.7d^{2}-816.9d+174.8$$ (13)

**Table 1:** Calculation results of samples.

Sample number	before normalization		after normalization		actual lifetime T/time	Criterion d
	mean/mΩ	fluctuation/mΩ	mean value	fluctuation		
**1**	5.81	1.68	1.00	0.62	18 514	0.90
**2**	5.78	1.91	0.99	0.70	1 540	0.92
**3**	5.22	2.72	0.90	1	1 965	0.93
**4**	5.38	2.11	0.93	0.77	31 150	0.88
**5**	5.39	2.40	0.93	0.88	12 247	0.91
**6**	5.16	2.11	0.89	0.77	40 804	0.86
**7**	5.31	0.75	0.91	0.27	46 018	0.78
**8**	5.45	1.07	0.94	0.39	44 312	0.81

**Fig 6:**
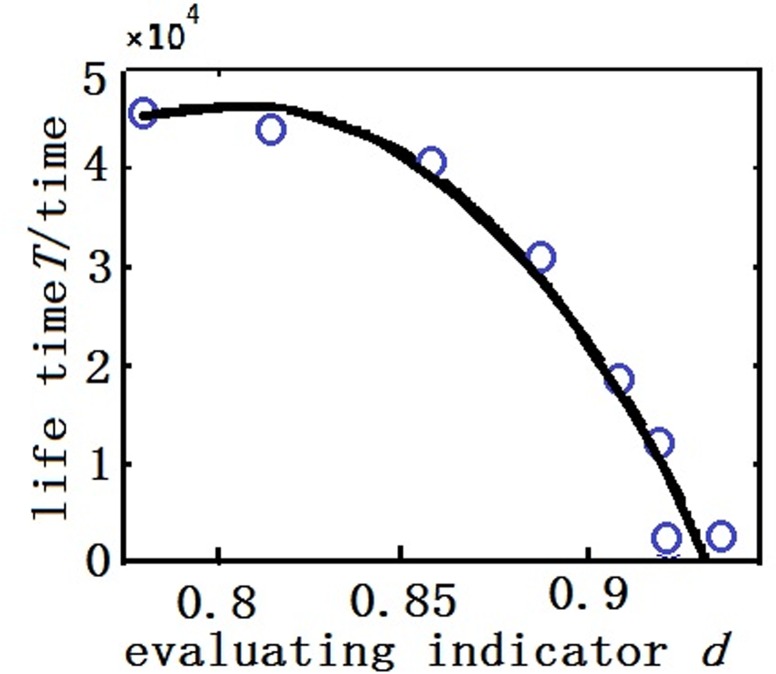
The relation between evaluation index and lifetime.

Fig 6 shows the fitting curve of the relationship between the criteria and actual lifetimes of all the samples.

The aforementioned estimation method was subsequently applied to estimate the initial contact resistance of an additional sample, and its parameter mean and stability before and after normalization can be seen from Table 2.

**Table 2:** Calculation results of a new sample.

	before normalization		after normalization		Evaluating indicator
	mean/mΩ	fluctuation/mΩ	mean	fluctuation	
**new sample**	5.38	2.25	0.93	0.82	0.8962

A whole lifetime test was conducted on the sample to estimate its actual lifetime, and (13) was used to derive its predicted lifetime. Comparison of predicted life and actual life can be seen from Table 3.

**Table 3:** Comparison of the values between actual life and predicted life.

actual life /time	predicted lifetime /time	absolute error/time	Relative error/%
25405	21500	3905	15.4

## 5. Conclusion

Initial parameter information indicates potential defects in electronics; higher levels of such defects suggest shorter lifetimes. This study proposed models for the relationships between the initial parameter information and lifetimes of several samples of an electronic device when the levels of benefit, cost, and moderate parameters were appropriate and stable. Parameter value and stability were quantified for small-sample modeling to model the relationship between the initial contact resistance and lifetime of several sample relays. The relative error between prediction lifetime obtained by prediction model and actual lifetime obtained by whole lifetime test is 15.4%. And the findings of this study indicate two conclusions:

1. The means of the initial parameter information derived using the probability-weighted average method denote the values of the parameters, and the difference between the extrema and end points on the fitting curve of the parameters represents the stability of the parameters. These quantitative analyses inform the lifetime prediction of electronics based on their initial parameter information.
2. When the sample size is limited, the relationship between criteria and lifetime that is established using the weighted distance method can be modeled to perform lifetime predictions. The lower the criteria are, the longer the predicted lifetime is.
